# A novel qPCR‐based technique for identifying avian sex: An illustration within embryonic craniofacial bone

**DOI:** 10.1002/dvg.23530

**Published:** 2023-06-24

**Authors:** Claire J. Houchen, Maria Bergman‐Gonzalez, Erin E. Bumann

**Affiliations:** ^1^ Department of Oral and Craniofacial Sciences, School of Dentistry University of Missouri‐Kansas City Kansas City Missouri USA

**Keywords:** chicken, duck, *HINTW* gene, quail, RT‐qPCR, sex differences, sex typing

## Abstract

Sex is a biological variable important to consider in all biomedical experiments. However, doing so in avian embryos can be challenging as sex can be morphologically indistinguishable. Unlike humans, female birds are the heterogametic sex with Z and W sex chromosomes. The female‐specific W chromosome has previously been identified in chick using a species‐specific polymerase chain reaction (PCR) technique. We developed a novel reverse transcription quantitative PCR (RT‐qPCR) technique that amplifies the W chromosome gene histidine triad nucleotide‐binding protein W (*HINTW*) in chick*,* quail*,* and duck. Accuracy of the *HINTW* RT‐qPCR primer set was confirmed in all three species using species‐specific PCR, including a novel quail‐specific *HINTW* PCR primer set. Bone development‐related gene expression was then analyzed by sex in embryonic lower jaws of duck and quail, as adult duck beak size is known to be sexually dimorphic while quail beak size is not. Trends toward sex differences were found in duck gene expression but not in quail, as expected. With these novel RT‐qPCR and PCR embryo sexing methods, sex of chick, quail, and duck embryos can now be assessed by either/both RNA and DNA, which facilitates analysis of sex as a biological variable in studies using these model organisms.

Sex is known to influence the development of human organs and organ systems, such as the skeletal system (Broere‐Brown et al., 2016; Hasselstrøm et al., 2006). It is therefore critical to analyze sex as a biological variable in both clinical and preclinical developmental studies. Further, analyzing sex as a biological variable is mandated by some funding bodies, such as the National Institutes of Health (NIH), in an effort to increase reproducibility and counteract overrepresentation of male animals, tissues, and cells in preclinical research (Clayton & Collins, 2014). Birds such as the chicken (*Gallus gallus*) have long been important developmental biology model organisms because they are experimentally convenient for observing and manipulating in ovo embryogenesis. A complication of using an avian developmental model is that sex is morphologically indistinguishable in most avian embryos, necessitating the use of a molecular technique to identify the sex of an avian embryo.

Humans have X and Y sex chromosomes with males being the heterogametic sex (Figure 1a), while birds have Z and W sex chromosomes with females being the heterogametic sex (Figure 1b). Determining bird sex has historically been complicated by our lack of understanding of the genetic mechanisms underlying avian sex determination, but recent evidence points to a dose‐dependent Z chromosome mode of sex determination in birds (Ioannidis et al., 2021; Smith, Roeszler, & Sinclair, 2009). There are instances when the presence or absence of a sex chromosome may not reflect the sex phenotype of an individual in many species, but the presence of a W chromosome in birds should usually indicate a female phenotype.

**FIGURE 1:**
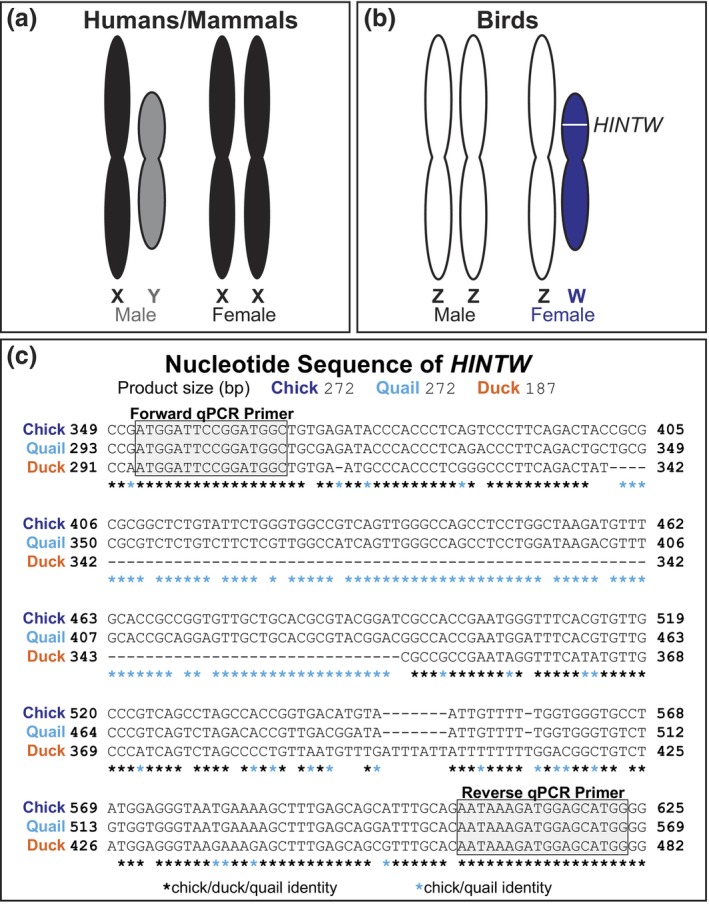
The female‐specific *HINTW* gene is conserved in chick, quail, and duck, and can be identified with a single novel *HINTW* RT‐qPCR primer set. (a) Humans have X and Y sex chromosomes with males being the heterogametic sex, while (b) birds have Z and W sex chromosomes with females being the heterogametic sex. The *HINTW* gene is located on the W chromosome and thus should only be present in female birds. (c) Our novel *HINTW* RT‐qPCR primers align to regions of the *HINTW* gene identical in chick, quail, and duck. The RT‐qPCR amplicon of these primers is 272 bp in female chick and quail but is 187 bp in female duck. RT‐qPCR, reverse transcription quantitative polymerase chain reaction.

Histidine triad nucleotide‐binding protein W (*HINTW*, sometimes called *WPKCI)* is a gene on the W chromosome (Figure 1b). *HINTW* likely does not play a role in sex determination, which aligns with the recent evidence suggesting avian sex is Z chromosome dose‐dependent (Ayers, Smith, & Lambeth, 2013; Ioannidis et al., 2021; Smith et al., 2009). Though likely not involved in sex determination, *HINTW* is a well‐established marker of female sex in chick cells and tissues as it is absent in male cells and tissues (Clinton, 1994; Nagai, Sezaki, Bertocchini, Fukuda, & Sheng, 2014). To our knowledge, no previous techniques have been described that use *HINTW* PCR in non‐chick bird species to identify sex during embryogenesis.

It is ideal for there to be a multitude of tools to identify avian sex because commonly used sex‐typing methods tend to only work in a subset of bird species, and it would be prudent in some cases to use multiple sex‐typing methods to verify the sex of an organism (Dawson et al., 2016; Griffiths, Double, Orr, & Dawson, 1998). To this end, this manuscript describes a novel reverse transcription quantitative polymerase chain reaction (RT‐qPCR) technique to amplify *HINTW* not only in chick, but also other bird models used in embryological studies such as quail (*Coturnix japonica*) and duck (*Anas platyrhynchos*). This single set of *HINTW* RT‐qPCR primers identifies sex of chick, quail, and duck using RNA which can then be used for other RNA‐based experimental analyses. Chick and quail are close evolutionary relatives and consequently their *HINTW* orthologs have high sequence identity and the same *HINTW* RT‐qPCR product size of 272 bp (Figure 1c). The *HINTW* RT‐qPCR primer set produces a 187 bp product for the less closely related duck (Figure 1c). We designed the forward and reverse *HINTW* primers which bind in areas where sequences were identical in all three avian species (Figure 1c). Chick and duck sex was validated by analyzing genomic DNA using established species‐specific PCR primers, while quail sex was validated using newly designed quail‐specific *HINTW* PCR primers. All PCR and RT‐qPCR primer sequences, amplicon sizes, species, and sources are listed in Table 1.

**TABLE 1:** Newly designed and previously published PCR and RT‐qPCR primers. Gene target, forward and reverse primer sequences, predicted amplicon size, species, and references, as appropriate, are listed

		Primer sequence (5′‐3′)	Predicted amplicon size (bp)	Species	References
**PCR**					
chick *HINTW*	Forward	CCCAAATATAACACGCTTCACT	450	Chick	Clinton (1994)
	Reverse	GAAATGAATTATTTTCTGGCGAC			
**quail *HINTW***	Forward	CATTTGAAGATTGTCGGCG	321	Quail	**This manuscript**
	Reverse	CCATGCTCCATCTTTATTGTGC			
*Z43B*‐W	Forward	CTTGAGACTAATTCCACTCC	263	Duck	Dawson, Dos Remedios, and Horsburgh (2016)
	Reverse	TTTACATGGCAGCTTGA			
*Z43B*‐Z	Forward	CTTGAGACTAATTCCACTCC	266 (chick/quail) and 271 (duck)	Chick/quail/duck	Dawson et al. (2016)
	Reverse	TTTACATGGCAGCCTGA			
**qPCR**					
***HINTW***	Forward	ATGGATTCCGGATGGC	272 (chick/quail) and 187 (duck)	Chick/quail/duck	**This manuscript**
	Reverse	CCATGCTCCATCTTTATT			
*GAPDH*	Forward	AAGCAGGACCCTTTGTTGG	155 (chick), 158 (quail), and 154 (duck)	Chick/quail/duck	Houchen et al. (2023)
	Reverse	ACAGATCAGTTTCTATCAGCCTCTC			
***RANK***	Forward	TTCAGGGACAAACAGCAGC	121 (quail and duck)	Quail/duck	**This manuscript**
	Reverse	GACGATGATGTCTCCCTTG			
*CTSK*	Forward	CTGGAGGGGCAGCTGAAGCG	124 (quail and duck)	Quail/duck	Houchen et al. (2023)
	Reverse	CGTATTCGAAGGCGTTGGTC			
***MEPE***	Forward	CCTCTTCTGCCTCTGCCT	111 (quail) and 108 (duck)	Quail/duck	**This manuscript**
	Reverse	CCTTTCAGCAGTATCTGGTGC			
***FGF23***	Forward	ATAATCACAGGTGTGAAGAGTGG	208 (quail and duck)	Quail/duck	**This manuscript**
	Reverse	GTGGTGGATTCATACCAGGG			
*MMP13*	Forward	GCTGGAGACAGAGATCCCAACCCA	139 (quail and duck)	Quail/duck	Ealba et al. (2015)
	Reverse	GCGGGTGCAGTCGCCAGAAA			
***OPN***	Forward	ATCCAGGCAGCATGCC	142 (quail and duck)	Quail/duck	**This manuscript**
	Reverse	AAGTGAGGCCAGGTCATTCTG			
*RUNX2*	Forward	TGGACCTTTCCAGACCAGCAGCA	162 (quail and duck)	Quail/duck	Ealba and Schneider (2013)
	Reverse	GGCAAGTTTGGGTTTAGCAGCGT			
*COL1A1*	Forward	CCCGACCCTAAGACAAAGAG	143 (quail and duck)	Quail/duck	Ealba et al. (2015)
	Reverse	GCTACTTACTGTCCTCTTCTCC			
*MMP9*	Forward	CGGCAGCCAAGAGCATGGTGA	179 (quail and duck)	Quail/duck	Ealba et al. (2015)
	Reverse	AGCTGGCCCCGTTGGCATTC			

*Note*: novel primers indicated by bold text.

Abbreviation: RT‐qPCR, reverse transcription quantitative polymerase chain reaction.

Male and female chicks were identified via PCR using the already‐validated chick PCR *HINTW* primers (Figure 2a; Clinton, 1994). *Z43B* primers specific to the Z chromosome (*Z43B*‐Z) were used as an internal and technical positive control for PCR since the Z chromosome is present in both males and females (Figure 2a; Dawson et al., 2016). Messenger RNA from those same individuals was tested in triplicate via RT‐qPCR using our novel *HINTW* primers. Female chick showed robust *HINTW* amplification, but *HINTW* products in male chick were undetected or amplified after cycle 35, which was considered a negative result (Figure 2b). The chick *HINTW* RT‐qPCR product in females was 272 bp long and melted at approximately 89 °C; no male RT‐qPCR product was detected by melt curve analysis (Figure 2c) or gel electrophoresis (Figure 2d). For RT‐qPCR, melt curve analysis, and gel electrophoresis, *GAPDH* served as an internal and technical positive control. This suggests our novel *HINTW* RT‐qPCR primer reliably sex‐types chick embryos.

**FIGURE 2:**
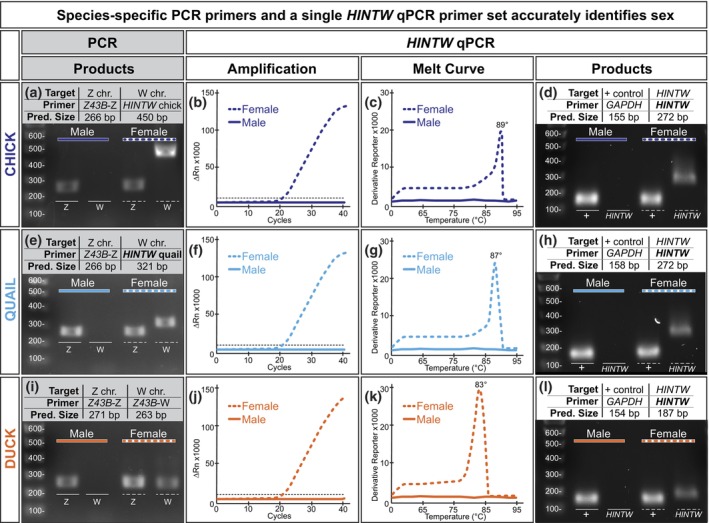
Species‐specific PCR primer sets and a single *HINTW* RT‐qPCR primer set accurately identifies sex. (a) Chick (in dark blue) DNA was analyzed by PCR using the Z chromosome‐specific *Z43B*‐Z primer set and the W chromosome‐specific *HINTW* chick‐specific primer set. Homogametic males display a Z chromosome band but no W chromosome band, while heterogametic females display both a Z and W chromosome band. (b) Messenger RNA was extracted from the same chick and was analyzed by RT‐qPCR using our novel *HINTW* RT‐qPCR primer set. Female chick RNA robustly amplified, while male chick RNA did not amplify or amplified after cycle 35, which is considered a negative result. (c) The melt curve summary graph displays a single 89 °C melt peak of the *HINTW* product in females. (d) RT‐qPCR products were run on a gel to compare predicted amplicon size to actual size. As indicated by amplification and melt curve analysis, no product was present in male chick while there was a single 272 bp female chick amplicon. *GAPDH* was the positive control. (e) Quail (in light blue) DNA was analyzed using the *Z43B*‐Z primers and our novel quail‐specific *HINTW* PCR primers. As in chick, both sexes had a Z chromosome band, but only females had a W chromosome band. (f) mRNA from the same quail was analyzed with our novel HINTW RT‐qPCR primers. Female quail showed robust amplification, while males had a negative result. (g) The melt curve summary graph demonstrates *HINTW*'s single 87 °C melt peak in female quail, and (h) the gel of the RT‐qPCR products shows the single *HINTW* amplicon was present in females only. (i) Similarly, duck (in orange) DNA was analyzed using the *Z43B*‐Z and W chromosome‐specific *Z43B*‐W primers. Males and females had Z chromosome bands, but only females had a W chromosome band. (j) Duck mRNA was analyzed by RT‐qPCR using the *HINTW* qPCR primer set. Female duck amplified robustly, while male duck had a negative result. (k) The melt curve summary graph demonstrates the single 83 °C melt peak of the *HINTW* product in females. (l) As in chick and quail, gel electrophoresis of RT‐qPCR products shows only female duck had a single *HINTW* amplicon. Sample size was *n* = 16–20/species for both PCR and RT‐qPCR. All amplification and melt curve graphs display data representative of all 16–20 biological replicates and technical triplicates. RT‐qPCR, reverse transcription quantitative polymerase chain reaction.

Neither the chick *HINTW* nor the duck *Z43B*‐W PCR primers successfully identified sex in quail samples (data not shown). In the absence of an established, reliable PCR‐based method for sex‐typing embryonic quail tissue, we designed a novel quail *HINTW* PCR primer set that amplified a 321‐bp segment of the *HINTW* gene in female quail but did not produce a product in males (Figure 2e). Messenger RNA from male and female individuals was analyzed via RT‐qPCR using our novel *HINTW* primer set. Female quail showed robust amplification, while male quail amplified after cycle 35 or did not amplify; this was consistent with what we predicted based on the chick data (Figure 2f). The quail RT‐qPCR product in females was 272 bp long with a melting peak at 87 °C, and no qPCR product was detected in male samples (Figure 2g,h). The same positive control PCR and RT‐qPCR primers were used for quail that were used for the chick PCR and RT‐qPCR experiments. These data suggest our novel *HINTW* RT‐qPCR primer reliably sex‐types quail embryos.

Male and female ducks were identified via PCR using already‐validated primers identifying the *Z43B* marker on the W chromosome (*Z43B*‐W; Figure 2i; Dawson et al., 2016). Messenger RNA of duck embryos was extracted and converted to cDNA and analyzed by RT‐qPCR using our *HINTW* primer set. Female duck *HINTW* amplified robustly, whereas male duck amplified after cycle 35 or did not amplify (Figure 2j). The *HINTW* RT‐qPCR product in female samples was 187 bp long and melted at 83 °C, while no RT‐qPCR product was detected in male samples (Figure 2k,l). The PCR and RT‐qPCR positive control primers used for duck were the same as were used for quail and chick. These data indicate that our novel RT‐qPCR *HINTW* primer set successfully sex‐types chick, quail, and duck.

The ability of this *HINTW* RT‐qPCR sexing technique to correctly identify *HINTW* in known female chick, quail, and duck samples was tested by comparing amplification in undiluted and serial 10‐fold diluted RNA samples (Figure S1). While *HINTW* amplification could be detected even in 100‐fold diluted RNA (Figure S1), it is important to use appropriate controls to ensure the integrity of positive and negative results. Refer to the flowchart in Figure S2 for assistance in experimental decision‐making and troubleshooting.

We applied our *HINTW* RT‐qPCR sexing technique to identify the sex of embryonic quail and duck using mRNA from the lower jaw. Adult duck beaks are longer in males, while quail beak size is not sexually dimorphic (Nudds & Kaminski, 1984). Beak shape and size are constructed from the underlying bone, therefore sex differences in adult duck beak length could be reflected in differential expression of genes related to jaw bone development in males and females.

Having established sex of all quail and duck individuals using lower jaw mRNA, expression of genes relevant to jaw development was then analyzed and compared across sexes. Multiple cell types contribute to bone formation and lower jaw bone length in quail and duck, including osteoclasts, osteocytes, and osteoblasts (Ealba et al., 2015; Hall et al., 2014; Houchen et al., 2023). Osteoclasts resorb bone and express genes such as receptor activator of nuclear factor κ B (*RANK*) and cathepsin K (*CTSK*). Osteoblasts, on the other hand, build bone and differentiate into osteocytes, which are cells that are housed in mineralized bone and use mechanical sensing to regulate the homeostasis of bone. Genes enriched in osteocytes include matrix extracellular phosphoglycoprotein (*MEPE*) and fibroblast growth factor‐23 (*FGF23*; Agoro et al., 2023; Dallas & Bonewald, 2010). Other osteoblast lineage cells play a role in bone development and function; pre‐osteoblasts express genes such as matrix metalloproteinase 13 (*MMP13*) and osteopontin (*OPN*). Additionally, genes such as runt‐related transcription factor 2 (*RUNX2*) and collagen type I alpha 1 chain (*COL1A1*) are expressed in osteolineage cells both at the osteoblast stage and differentiated osteocyte stage (Agoro et al., 2023). These eight genes constitute only a fraction of the genes involved in bone development and were selected for this analysis as they represent multiple cell types known to be essential to bone development, as well as represent various stages of differentiation.

We analyzed expression of genes related to bone development in lower jaw tissue at Hamburger and Hamilton (HH) Stage 39, when the jaw is largely calcified and there is active bone resorption by osteoclasts, relative to expression at stage HH33, when bone mineralization is just beginning and there are no active osteoclasts in the lower jaw bone (Ealba et al., 2015; Hamburger & Hamilton, 1951; Houchen et al., 2023). Osteoclast *RANK* expression was mildly but significantly higher in male duck compared to female duck (*n* = 3–4/stage/sex, *p* = .03; Figure 3a). While expression of osteoclast genes *CTSK* (Figure 3b) and matrix metalloproteinase 9 (*MMP9*; data not shown) was similar in male and female duck, osteocyte *MEPE* expression was also significantly higher in male duck compared to female duck (*p* = .03; Figure 3c). Expression of another osteocyte gene, *FGF23*, was not statistically significantly different in male and female ducks (Figure 3d). Genes expressed in pre‐osteoblasts and osteolineage cells, such as *MMP13*, *OPN*, *RUNX2*, and *COL1A1*, did not differ between male and female ducks (Figure 3e–h). These data suggest there might be sex differences in duck lower jaw development underlying the beak sexual dimorphism seen in adult duck; however, these data are not meant to posit any particular bone mechanism in the duck embryos is the cause of the adult duck beak sexual dimorphism. Further future studies will be needed to identify the specific molecular mechanisms controlling sexual dimorphism in duck embryos.

**FIGURE 3:**
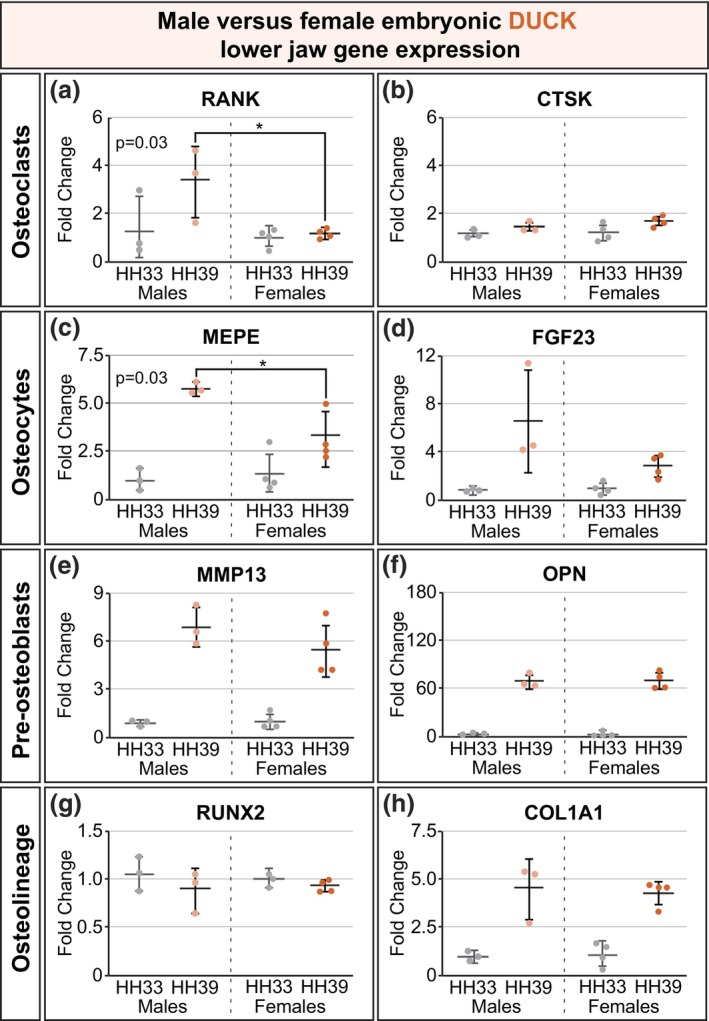
Expression of genes involved in embryonic bone development indicates possible sex differences in duck. (a, b) Expression of *RANK*, a gene enriched in osteoclasts, differed significantly between male and female ducks (*p* = .03), but another gene expressed in osteoclasts *CTSK* did not. (c) *MEPE* expression is enriched in osteocytes; *MEPE* expression also differed significantly between male and female ducks (*p* = .03). (d) Another osteocyte gene, *FGF23* expression was not statistically significantly different in males and females. (e) *MMP13* and (f) *OPN* are genes with enriched expression in pre‐osteoblasts, and neither indicates sex differences in gene expression. Similarly, (g) *RUNX2* and (h) *COL1A1* are genes expressed in osteolineage cells both at the osteoblast stage and at the osteocyte stage, and neither indicates differential expression in males and females. The sex differences indicated by these data aligns with known sexual dimorphism in adult duck beaks. Graphs show mean fold change ± standard deviation with all data points shown. Gray points indicate HH33 individuals, light orange points indicate HH39 males, and dark orange points indicate HH39 females; male HH39 fold change gene expression versus female HH39 fold change gene expression was compared by an unpaired two‐tailed Student's *t* test. *p < .05, *n* = 7/stage/species.

Gene expression related to bone development in the lower jaw was similarly analyzed in embryonic quail at HH33 and HH39. None of the genes analyzed (*RANK*, *CTSK*, *MEPE*, *FGF23*, *MMP13*, *OPN*, *RUNX2*, and *COL1A1*) in quail showed sex differences in expression (Figure 4a–h). Nor did expression of *MMP9* differ by sex (data not shown). This aligns with there being no documented sexual dimorphism in adult quail beaks. Gene expression analysis in Figures 3 and 4 included *n* = 3–4 individuals/sex/species/stage and is meant to exemplify the utility of the single set of *HINTW* RT‐qPCR primers to sex quail and duck embryos. Increasing the sample size for the gene expression analyses would increase the statistical power of these tests, and our data depict trends toward the presence or absence of sex differences in quail and duck jaw development gene expression rather than conclusive proof of sex differences.

**FIGURE 4:**
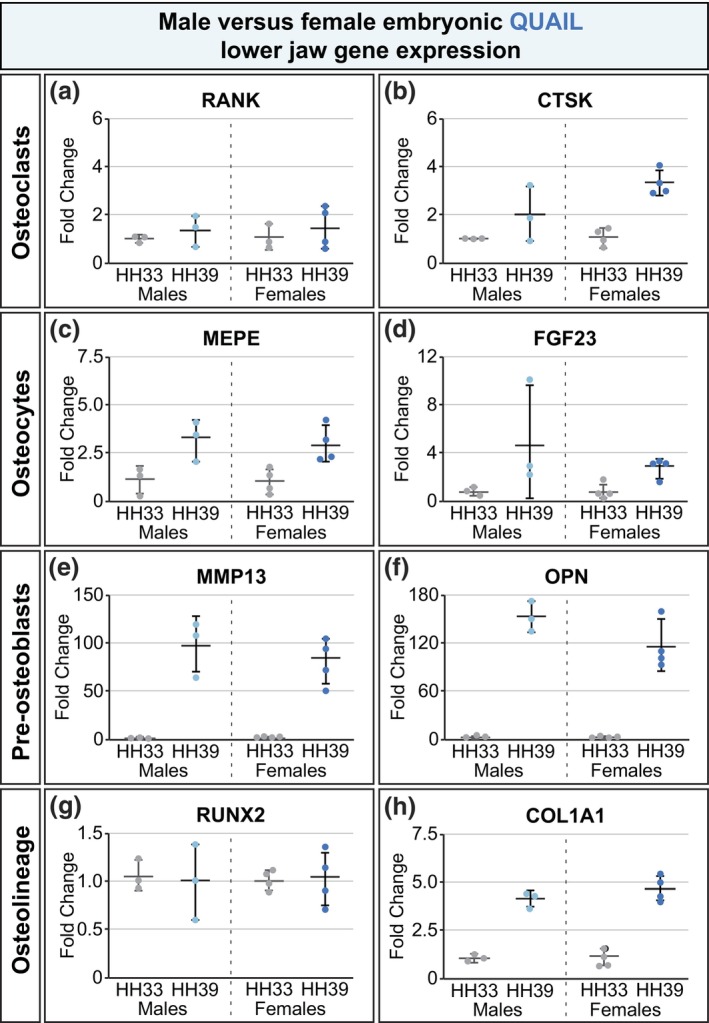
Expression of genes involved in embryonic bone development does not signify sex differences in quail. Though they represent multiple bone‐relevant cell types, none of the genes analyzed (*RANK*, *CTSK*, *MEPE*, *FGF23*, *MMP13*, *OPN*, *RUNX2*, and *COL1A1*) were expressed differently in male and female quail (a–h). This reflects that in contrast to duck, quail skeletons and beaks are not sexually dimorphic in adult specimens. Graphs show mean fold change ± standard deviation with all data points shown. Gray points indicate HH33 individuals, light blue points indicate HH39 males, and dark blue points indicate HH39 females; male HH39 fold change gene expression versus female HH39 fold change gene expression was compared by an unpaired two‐tailed Student's *t* test. *p < .05, *n* = 7/stage/species.

In summary, our novel RT‐qPCR *HINTW* technique can effectively identify sex of avian embryos in several species using a single primer set, which provides an RNA‐based strategy for accomplishing the sometimes‐challenging task of sex‐typing birds. Additionally, this RNA‐based sexing technique facilitates consideration of sex as a variable both before conducting expensive RNA analysis (such as RNA‐seq) and during data analysis in studies using birds. This RT‐qPCR *HINTW* technique is a novel RNA‐based strategy for sex typing chick, quail, and duck. We also described a novel PCR *HINTW* technique to identify sex of quail embryos, therefore the sex of all three of these species (chick, quail, and duck) can now be identified using either/both DNA‐ and RNA‐based techniques. We demonstrated the utility of this tool for considering sex as a biological variable by analyzing the expression of genes related to bone development in embryonic quail and duck and found there might be sex differences in duck lower jaw development underlying the sexual dimorphism seen in adult duck beak length.

## 1 METHODS

### 1.1 Use of avian embryos

Fertilized eggs of chick (*G. gallus*), Japanese quail (*Coturnix coturnix japonica*), and white Pekin duck (*A. platyrhynchos*) were purchased from AA Lab Eggs (Westminster, CA) and incubated at 37 °C in a humidified chamber. Eggs were kept in the incubator until they reached embryonic stages appropriate for analysis. For all procedures, we adhered to accepted practices for the humane treatment of avian embryos as described in S3.4.4 of the AVMA Guidelines for the Euthanasia of Animals: 2020 Edition (Leary et al., 2020). Embryos were staged using the HH staging system, a well‐established standard that was originally developed for chicks (Hamburger & Hamilton, 1951). While separate staging systems exist for duck and quail, these embryos can also be staged via the HH system (Ainsworth, Stanley, & Evans, 2010). Eggs were windowed and external morphological characteristics were used to determine staging. Accurate staging was necessary to ensure proper and consistent timing of collection at HH33 or HH39 for RT‐qPCR analysis.

### 1.2 Developing HINTW PCR primers for quail

Quail‐specific *HINTW* PCR primers were developed using Primer‐BLAST (Ye et al., 2012). The input PCR template *Coturnix coturnix japonica Wpkci* (*HINTW*) was retrieved from ENA (AB033881.1; Hori, Asakawa, Itoh, Shimizu, & Mizuno, 2000). The forward primer sequence was 5′‐3′ CATTTGAAGATTGTCGGCG (start: 247, stop: 265) and the reverse primer sequence was 5′‐3′ CCATGCTCCATCTTTATTGTGC (start: 567, stop: 546). The predicted product length was 321 bp, and in silico analysis using Primer‐BLAST predicted no off‐target products.

### 1.3 Identification of embryo sex using PCR

Genomic DNA was extracted from chick, duck, and quail skin tissue (*n* = 16–20/species) using Extract DNA Prep for PCR (QuantaBio 95091) following the manufacturer's protocol, and extracts were stored at −20 °C. DNA concentration and quality were assessed by a Nanodrop 2000 spectrophotometer (Thermo Fisher Scientific, Waltham, MA). All samples were diluted to 200–500 ng/μL in ddH_2_O, and DNA was considered pure at a 260/280 ratio of 1.8 ± 0.1. Reactions were performed in 25 μL volumes in a 0.2‐mL tube containing 12.5 μL REDTaq® ReadyMix™ PCR Reaction Mix (Sigma‐Aldrich R2523), 10.5 μL ddH_2_O, 0.5 μL forward primer, and 0.5 μL reverse primer. All PCR primers are listed in Table 1. Two reactions were run per individual: one reaction targeting the W chromosome (quail *HINTW* primers, chick *HINTW* primers, or *Z43B*‐W primers for duck) and one reaction targeting the Z chromosome as an internal and technical control (*Z43B*‐Z primers for all species).

Both the quail and chick *HINTW* PCR reactions used the following thermocycling parameters: 98 °C for 2 min; followed by 35 cycles of 98 °C for 10 s, 56 °C for 30 s, and 68 °C for 40 s; then 68 °C for 5 min. The *Z43B‐*Z and *Z43B‐*W PCR reactions used the following thermocycling parameters: 94 °C for 15 min; followed by 45 cycles of 94 °C for 30 s, 54 °C for 30 s, and 72 °C for 1 min; then 72 °C for 10 min. Products were then loaded in duplicate in 1.2% agarose gels containing MIDORI Green Advance Safe DNA Stain (Bulldog Bio MG04), and the gels were run in 1× TBE running buffer at 100 V for 90 min. The gel was then imaged using the Azure c400 Gel Imaging System (Azure Biosystems, Dublin, CA). Primer specificity was assessed by comparing actual band size to predicted band size, which are listed in Table 1. Presence of a band in the lane corresponding to a W chromosome primer (quail *HINTW*, chick *HINTW*, or *Z43B*‐W for duck) was considered indicative of a female, and presence of a Z chromosome band for the same individual was used as a positive control.

### 1.4 HINTW RT‐qPCR primer design

A *HINTW* RT‐qPCR primer set effective in chick, duck, and quail was developed using Primer‐BLAST (Ye et al., 2012). The input mRNA templates were: chick GenBank NM_204688.2 (Smith et al., 2009), quail ENA AB033881.1 (Hori et al., 2000), and duck ENA AB033883.1 (Hori et al., 2000). The forward primer sequence was 5′‐3′ ATGGATTCCGGATGGC and the reverse primer sequence was 5′‐3′ CCATGCTCCATCTTTATT. The predicted product length was 272 bp for chick and quail and 187 bp for duck.

Primer‐BLAST was also used to create four of the jaw bone development‐related genes analyzed in Figures 3 and 4: *RANK*, *MEPE*, *FGF23*, and *OPN*. The RT‐qPCR primers for *CTSK* originate from Houchen et al. (2023), the primers for *MMP9*, *MMP13*, and *COL1A1* originate from Ealba et al. (2015), and the *RUNX2* primers originate from Ealba and Schneider (2013). Primer sequences, predicted amplicon sizes, species, and primer sources are listed in Table 1.

### 1.5 HINTW and jaw bone development‐related gene expression analysis

Reverse transcription quantitative polymerase chain reaction (RT‐qPCR) was used to analyze stage‐ and species‐specific jaw bone development‐related mRNA expression, as well as presence or absence of *HINTW* mRNA expression (Bustin et al., 2009). Total RNA was isolated from *n* = 16 to 20/species chick, quail, and duck embryos at HH39 for initial *HINTW* RT‐qPCR primer testing and from an additional *n* = 7/stage chick, quail, and duck whole lower jaws at HH33 and HH39 for bone gene expression analysis using an RNAeasy column purification kit (Qiagen, Valencia, CA; Ealba & Schneider, 2013). A Nanodrop 2000 spectrophotometer (Thermo Fisher Scientific, Waltham, MA) was used to assess concentration and purity of RNA. RNA was stored at −80 °C. Approximately 250 ng of total RNA was converted to cDNA in a 20‐μL reverse transcription reaction using the Applied Biosystems™ High‐Capacity cDNA Reverse Transcription Kit (Thermo Fisher Scientific, Waltham, MA). The reaction involved: 25 °C for 10 min; 37 °C for 120 min; 85 °C for 5 min; then 4 °C hold in a C1000 Touch Thermal Cycler (Bio‐Rad, Hercules, CA). cDNA was stored at −20 °C.

RT‐qPCR was performed with a StepOnePlus™ Real‐Time PCR System (Thermo Fisher Scientific, Waltham, MA). Forward and reverse primers, 2 μL of cDNA, RNase‐free dH2O, and iQ SYBR‐Green Supermix (Bio‐Rad, Hercules, CA) were manually mixed in a 20 μL reaction to amplify the cDNA of interest. Samples were run in triplicate on white hard‐shell 96‐well plates (Bio‐Rad, Hercules, CA). The protocol was: 95 °C for 3 min; then 40 cycles of 95 °C for 10 s, 60 °C for 30 s, and a plate read; followed by 95 °C for 10 s; finished by melt curve for 60–90 °C for 5 s at each 0.5 °C with a plate read. Melt curves were analyzed to confirm specificity of primer. RT‐qPCR products that were amplified after 35 cycles were considered false positives. Following RT‐qPCR, products were run on a 1% agarose gels containing MIDORI Green Advance Safe DNA Stain (Bulldog Bio MG04), and the gels were run in 1× TBE running buffer at 100 V for 90 min. The gel was then imaged using the Azure c400 Gel Imaging System (Azure Biosystems, Dublin, CA). Predicted amplicon size was compared to band size; this was used as a primer specificity check in addition to melt curve analysis.

All RT‐qPCR primer sequences and predicted amplicon sizes are listed in Table 1. Primers were designed for use in both quail and duck using Primer‐BLAST software (Ye et al., 2012). Expression levels of all jaw development‐related genes were normalized to expression of the reference gene glyceraldehyde 3‐phosphate dehydrogenase (*GAPDH*). Expression was checked to make sure amplification efficiencies were equal among samples. Fold changes were calculated using the delta–delta C(t) method (Livak & Schmittgen, 2001). To assess relative fold changes between stages, expression at HH39 is relative to expression at HH33.

To assess the ability of the RT‐qPCR *HINTW* method to detect low concentrations of *HINTW*, 10‐fold dilutions of 2.5 ng/μL female chick, quail, and duck RNA were tested in triplicate with the RT‐qPCR *HINTW* primers. The standard curve was created by plotting cycle of amplification versus RNA concentration.

### 1.6 Statistical analysis

RT‐qPCR data in Figures 3 and 4 are shown by mean fold change ± standard deviation with all data points shown. Male HH39 fold change gene expression versus female HH39 fold change gene expression was compared by an unpaired two‐tailed Student's *t* test with a significance cutoff of *p* < .05. The standard curve in Figure S1 was analyzed by simple linear regression.

## Supporting information


**FIGURE S1.** HINTW can be detected in highly diluted RNA samples. Tenfold dilutions of 2.5 ng/μL female chick, quail, and duck RNA/cDNA in triplicate were tested with the RT‐qPCR *HINTW* primers. (a, b) Each 10‐fold dilution resulted in amplification occurring three to four cycles later than the previous dilution, and true positives could be reliably detected down to 1% RNA concentration. At 0.1% RNA concentration, some samples become false negatives, and at 0.01% RNA concentration, all samples became false negatives and read as “undetected.”
**FIGURE S2.** Flowchart to assist with experimental decision‐making for using techniques described in this article to identify sex of chick, quail, and/or duck embryos. This flowchart can be used to guide experimental design and decision‐making when using RNA (in green) or DNA (in purple) to identify sex of chick, quail, and/or duck embryos. All primers mentioned in the flowchart can be found in Table 1.

## Data Availability

The data that support the findings of this study are available from the corresponding author upon reasonable request.
