# Supplementary material for: A novel qPCR‐based technique for identifying avian sex: An illustration within embryonic craniofacial bone
Source: Genesis. 2023 Jun 24;62(1):e23530. doi: 10.1002/dvg.23530 (PMC11457736; doi:10.1002/dvg.23530)

Supplemental Figure 1

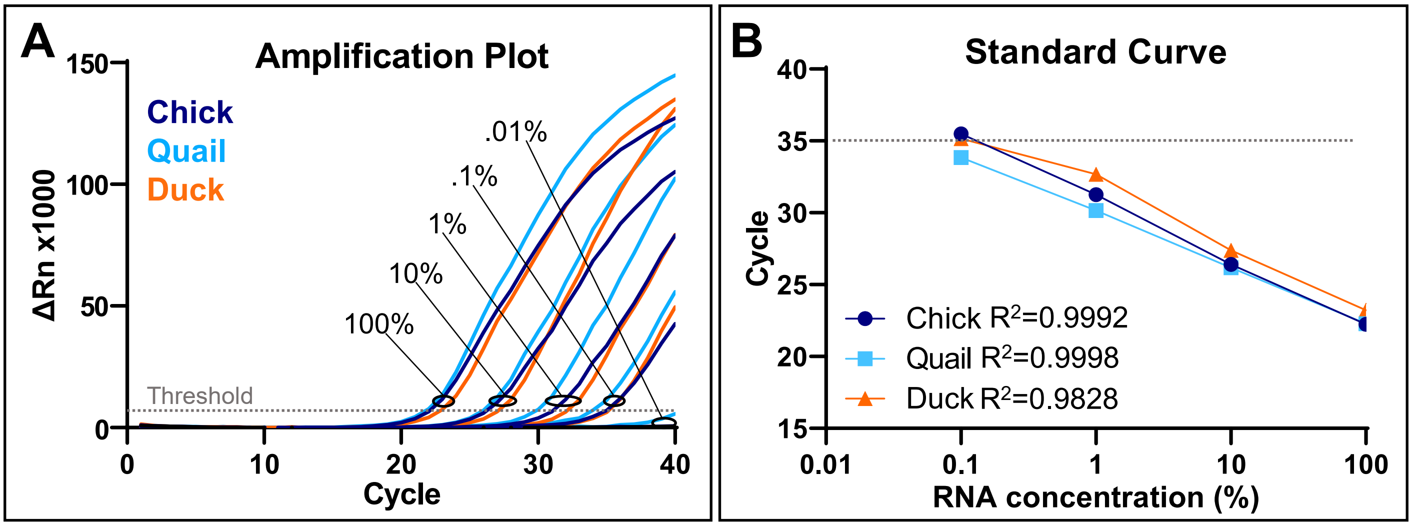

Supplemental Figure 2

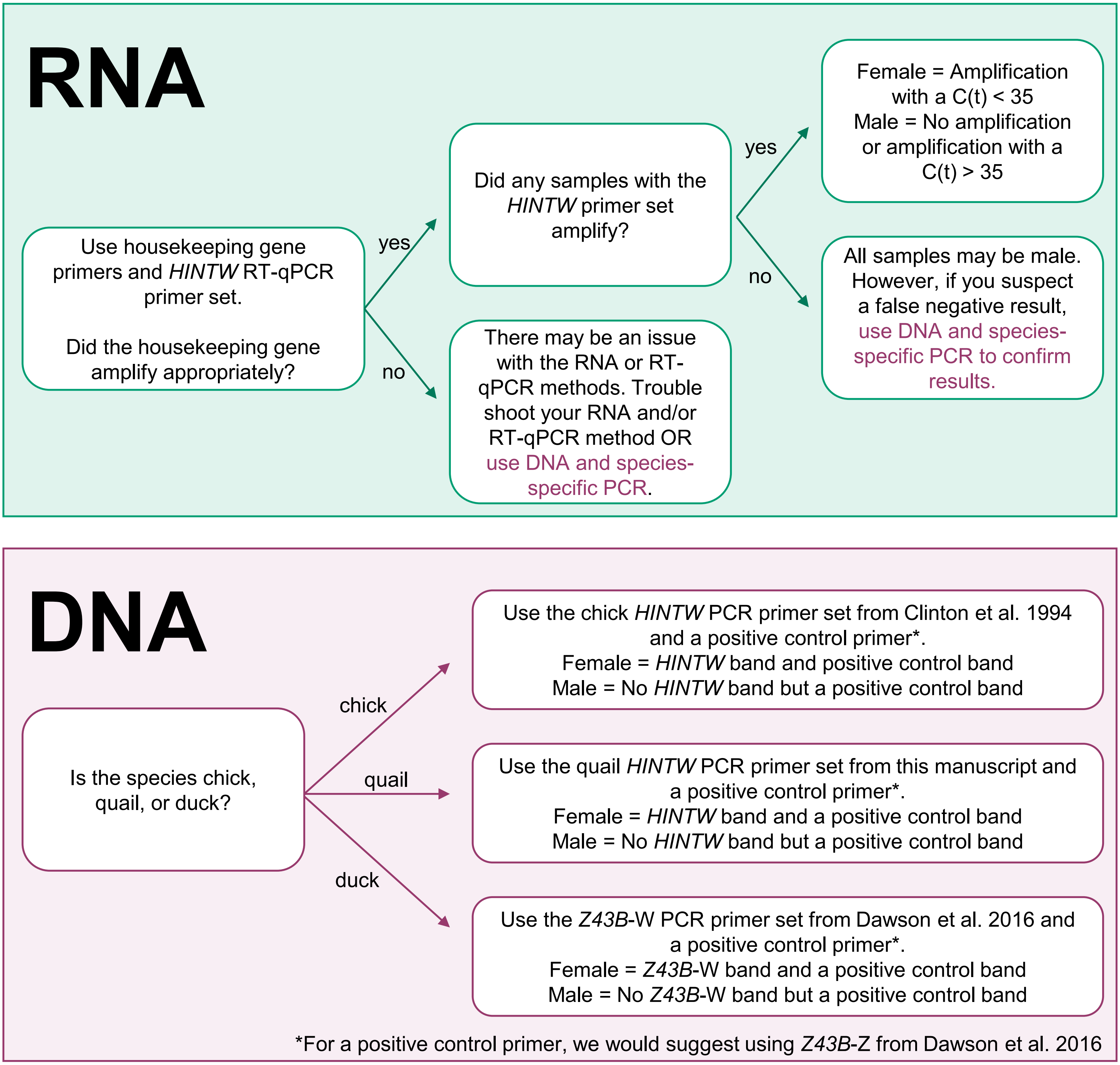

Supplement: Supplementary file 1 — FIGURE S1. HINTW can be detected in highly diluted RNA samples. Tenfold dilutions of 2.5 ng/μL female chick, quail, and duck RNA/cDNA in triplicate were tested with the RT‐qPCR HINTW primers. (a, b) Each 10‐fold dilution resulted in amplification occurring three to four cycles later than the previous dilution, and true positives could be reliably detected down to 1% RNA concentration. At 0.1% RNA concentration, some samples become false negatives, and at 0.01% RNA concentration, all samples became false negatives and read as “undetected.” FIGURE S2. Flowchart to assist with experimental decision‐making for using techniques described in this article to identify sex of chick, quail, and/or duck embryos. This flowchart can be used to guide experimental design and decision‐making when using RNA (in green) or DNA (in purple) to identify sex of chick, quail, and/or duck embryos. All primers mentioned in the flowchart can be found in Table 1. [file DVG-62-e23530-s001.pdf]
